# Child maltreatment and substance use: associations with alcohol use, nicotine use, and quality of life in a Norwegian population sample

**DOI:** 10.3389/fpubh.2026.1821179

**Published:** 2026-05-15

**Authors:** Frida Grimsland, Karin Berle Gabrielsen, Thomas Clausen, Siri H. Haugland, John-Kåre Vederhus

**Affiliations:** 1 Addiction Unit, Sørlandet Hospital Trust, Kristiansand, Norway; 2 Norwegian Center for Addiction Research (SERAF), University of Oslo, Oslo, Norway; 3 Department of Psychosocial Health, University of Agder, Grimstad, Norway

**Keywords:** child maltreatment, nicotine use, quality of life, smoking, snus

## Abstract

**Background:**

Child maltreatment (CM) is a significant public health concern with long-term consequences for the child, including an increased likelihood of engaging in risky health behaviors, such as substance use, as well as impaired quality of life (QoL). The relationship between CM and specific substance use patterns, particularly the use of nicotine products such as Swedish snus, remains understudied. We investigated the relationship between CM and the use of nicotine and alcohol, as well as whether use of these substances mediates the association between CM and adulthood QoL.

**Methods:**

This study utilized data from the Norwegian Counties Public Health Survey for Agder in 2023. Latent regression analysis was employed to examine the association between CM and QoL, as well as potential mediated associations involving substance use, while controlling for sociodemographic factors.

**Results:**

Nicotine use in the past year was significantly more prevalent among individuals with a history of CM compared to the group with no history of CM (35.8% vs. 25.6%, *p* < 0.001). Both smoking (24.3% vs. 15.6%, *p* < 0.001) and snus use (17.6% vs. 13.3%, *p* < 0.001) were more common in the CM group, with differences remaining significant after adjustment for sociodemographic factors. No significant differences were found in alcohol use across various measures, including frequency of use, AUDIT-C scores, and binge drinking. Latent regression analysis showed that CM had an overall negative association with QoL [β = −0.61; 95% confidence interval (CI) −0.71, −0.51; *p* < 0.001]. A significant mediated association involving nicotine use was also observed (β = −0.12; 95% CI −0.16, −0.07; *p* < 0.001).

**Conclusion:**

CM is associated with a higher prevalence of smoking and snus use, which are negatively associated with QoL. The findings suggest that targeted efforts to support QoL among individuals with a history of CM, including attention to nicotine use, may be relevant within broader strategies addressing living conditions and psychological wellbeing.

## 1 Introduction

Adverse childhood experiences (ACEs) involve a wide range of experiences comprising household dysfunction and various forms of child maltreatment (CM), including physical, sexual, and emotional abuse (1). Prevalence of CM differs substantially by type, gender, and continent, but the median prevalence across continents is 2.6%−28.8% for child sexual abuse, 6.7%−60.2% for child physical abuse, and 6.2%−60.9% for child emotional/psychological abuse (2). In Norway, the prevalence of physical, emotional, and sexual abuse in childhood was recently estimated to be 19%, 18%, and 6%, respectively (3).

ACEs pose significant risks to both individual and public health, with well-established associations with physical and psychiatric morbidity in adulthood (4). In addition to impaired health, ACEs are associated with reduced quality of life (QoL) in adulthood, which may serve as a comprehensive proxy for overall health status (5–7). One mechanism by which ACEs influence health negatively is the increased tendency for risky health behaviors, including the use of psychoactive substances (8, 9). A moderately strong association has been found between ACEs and smoking (9), and an association with the use of tobacco has also been found in regions where smokeless tobacco is the predominant form of tobacco used (10). The association between ACEs and problematic alcohol use has generally been considered to be strong (9). Nonetheless, some recent studies have reported inconsistent or weak associations between ACEs and alcohol consumption (11–15).

The harmful effects of tobacco on health are well established. Smoking can be linked to disease in almost every organ system (16). Active smokers increase their risk of premature death by threefold (17). Regarding smokeless tobacco, health outcomes differ by product and region (18). Swedish snus in particular is less studied, but there is evidence of an association with an increased risk of pancreatic and esophageal cancer, a potential link to stomach and rectal cancer, and an indication of increased overall and cancer-specific mortality following cancer diagnosis (19). Furthermore, snus use has been associated with hypertension, mortality after stroke and myocardial infarction, type 2 diabetes, and metabolic syndrome (20). Encouragingly, the prevalence of global tobacco use has decreased by one-third over the past two decades (21). Yet, the number of people using tobacco globally is high; a total of 1.20 billion users aged ≥15 years was expected in 2025 (22). In Norway, there has been a steady decline in smoking, from a prevalence slightly greater than 40% in 1973 to less than 10% in 2022 (23). The global prevalence is less certain for smokeless tobacco, but the estimate is at least 362 million adult users (age ≥15 years) of smokeless tobacco globally (22). In Norway, daily use of smokeless tobacco, primarily snus, now exceeds daily smoking (24). Over the past decade, snus use has surged nearly twofold, reaching 16% daily users in 2023 (24).

Similar to nicotine use, alcohol use poses significant risks to health. Acute intoxication can lead to life-threatening conditions and, over time, alcohol use can cause addiction and impact mental and physical health, including an increased risk of a variety of cancers and chronic diseases (25, 26). Global alcohol consumption has decreased overall, but alcohol is still the most used psychoactive substance of significant public health importance in the world (27). In Norway, there are social inequalities in smoking and alcohol consumption, with lower educational status linked to higher use (28, 29). For snus, however, the association with education is less clear (30).

Violence against children remains a neglected research field within public health (31). Furthermore, the association between specific ACEs and the use of particular tobacco products is under-investigated (32). Examining whether ACEs, a known risk factor for nicotine use, remain significant in a population with shifting tobacco use patterns may yield valuable insights into the field. Given the increasing use of snus, it is important to investigate whether established risk factors for smoking also apply to snus. Regarding alcohol, we investigated whether our data support the widely held assumption that ACEs are associated with increased alcohol consumption or if it contributes to the inconsistent findings in the literature on this relationship (9, 12, 13).

The aim of the current study was to investigate the prevalence of CM and its associations with the use of nicotine and alcohol, as well as the impact on QoL, in a general population sample. We examined group differences in substance use between individuals with and without a history of CM. For substances with significant group differences, latent regression analysis was conducted to explore the direct and indirect association between CM and QoL while adjusting for sociodemographic variables.

## 2 Method

### 2.1 Data source and participants

The present study utilized data from a regional Norwegian public health survey conducted in Agder County, Norway, in 2023. The survey was part of the ongoing Norwegian Counties Public Health Surveys (NCPHS) administered by the Norwegian Institute of Public Health (NIPH) (33). The survey covers a broad range of topics, including childhood experiences, health behaviors, and psychosocial functioning in adulthood, to assess trends over time and provide insights into the adult population on topics relevant to public health planning at the county and municipals levels (34). A stratified random sample of adults aged ≥18 years was drawn from the National Population Register, targeting all residents in Agder. Eligible participants were contacted via SMS and secure digital mail (Digipost) and invited to complete the questionnaire. Of the 57 891 residents invited to participate, a total of 18 517 individuals completed the survey, yielding a participation rate of 32%.

### 2.2 Measures

Basic sociodemographic variables included in the survey were age, sex, and educational attainment.

#### 2.2.1 Childhood Maltreatment (CM)

CM was assessed by asking participants whether they had experienced abuse or violence by a close caregiver during childhood. Response options were “yes” or “no.” Those who responded “yes” were asked to indicate the type(s) of maltreatment they had experienced by choosing from “emotional abuse” (e.g., being demeaned, threatened, humiliated, or emotionally harmed), “physical abuse” (e.g., being subjected to physical harm or pain), or “sexual abuse” (e.g., forced sexual acts or unwanted sexual advances). Participants could also select “other” or “prefer not to answer.” For the latent regression model, emotional, physical, and sexual abuse were used as indicators of the latent CM construct. These subtypes correspond to direct forms of maltreatment and align with the core maltreatment categories in the ACE framework. Responses in the “other/prefer not to answer” category were retained for descriptive reporting but were not included in the latent CM construct. This category could not be incorporated as an additional indicator because it did not represent a clearly defined subtype and did not form meaningful associations with the three specific CM indicators required for latent variable modeling.

#### 2.2.2 Substance use

Nicotine use was assessed by separate items on cigarette smoking and the use of snus in the past 12 months. Response options for each product included “never,” “occasionally,” and “daily/near-daily use.” We also used a combined variable representing any nicotine use. For the latent regression analysis, we modeled nicotine use as a latent construct, based on binary (yes/no) indicators for each product (smoking and snus).

Alcohol consumption was measured using the Alcohol Use Disorders Identification Test–Consumption (AUDIT-C), a three-item screening tool widely used to detect hazardous and harmful drinking. Response options included drinking frequency over the past 12 months, number of drinks on a typical drinking day, and self-reported binge drinking, defined as consuming 6 or more standard units of alcohol on a single occasion. Each item was scored on a scale from 0 to 4, with a maximum total score of 12. We used an AUDIT-C score cut-off of 4 for women and 5 for men, as these scores have been found to be the most accurate for identifying risky drinking (35). However, given concerns that this threshold may overestimate prevalence, a stricter cut-off of 8 was also applied (35).

#### 2.2.3 Quality of life

QoL was measured using the Essential QoL-3 (EQoL-3) scale, which assesses perceived satisfaction with life, happiness, and meaningfulness. Participants rated each item on a scale of 0 to 10, with higher scores indicating greater satisfaction, happiness, and perceived meaningfulness. Scores were averaged to provide an overall QoL score (36). The reliability of the scale was excellent with a Cronbach's alpha of 0.86.

### 2.3 Statistical analysis

Descriptive statistics were used to characterize the sample and compare respondents with and without a history of CM. Group differences in nicotine and alcohol use were analyzed using chi-squared tests and logistic regression models adjusted for age, gender, and education. These analyses were carried out using IBM SPSS Statistics, version 30.0.

To investigate the relationships between CM and QoL, including potential mediation by nicotine use, we performed a latent regression analysis using structural equation modeling in Mplus version 8.6 (37, 38). CM and nicotine use were specified as latent constructs with their respective indicators, and QoL was modeled as a latent factor based on the ordinal indicators. Modeling smoking and snus use as a latent nicotine-use construct was preferred over treating these indicators as separate observed variables. The two behaviors shared substantial variance, and the latent factor approach provides a more coherent representation of overall nicotine involvement while reducing measurement error (39).Given that the key indicators were binary, models were estimated using the weighted least squares mean and variance adjusted (WLSMV) estimator, which is recommended for SEM with categorical data (39). Model fit was assessed using standard fit indices; root mean square error of approximation (RMSEA) ≤ 0.08 and comparative fit index (CFI) ≥0.90 indicated acceptable fit (40). Full information maximum likelihood estimation was used to handle missing data. All reported coefficients are unstandardized (β) with their 95% confidence interval (CI). *P*-values < 0.05 were considered significant.

## 3 Results

The mean age of respondents was 51.3 years (standard deviation, 16.3 years), the majority were women (56%), and roughly 4 in 10 had completed a bachelor's degree or higher (Table 1). One in 10 participants reported having experienced CM. The prevalence by subtype was 7.2% for physical abuse, 5.5% for emotional abuse, and 1.6% for sexual abuse.

**Table 1:** Characteristics of the sample population (*N* = 18 517).

Variable	*n* (%)
Sex, female	10 316 (55.7)
Education level (*N* = 18 395)	
- Primary and secondary school (up to 10 years of education)	1,463 (8.0)
- High school (up to 13 years of education)	8,959 (48.7)
- University college or university (bachelor's degree or greater)	7,973 (43.3)
Experienced childhood maltreatment (*N* = 18 334)	1,842 (10.0)
- Physical abuse	1,329 (7.2)
- Emotional abuse	1,021 (5.6)
- Sexual abuse	295 (1.6)
- Other or do not wish to specify	137 (0.7)
Nicotine use, current (*N* = 18 386)	4,892 (26.6)
- Smoking (*N* = 18 449)	3,045 (16.5)
- Snus (*N* = 18 419)	2,525 (13.7)
Alcohol use (*N* = 18 216)	
- Any alcohol use last 12 months	15 336 (83.4)
- AUDIT-C gender-specific thresholds^a^	5,835 (31.7)
- AUDIT-C ≥8	621 (3.4)
- Alcohol binge weekly or more (*N* = 18 271)	635 (3.5)

^a^Gender-specific cut-offs for the AUDIT-C sum score were 5 for men and 4 for women.

Nicotine use was significantly more common among individuals with a history of CM compared to those with no history of CM across all categories (Table 2). In the past 12 months, 35.8% of participants in the CM group reported use of a nicotine product compared to 25.6% of participants in the no-CM group (*p* < 0.001). For specific products, smoking was more prevalent in the CM group than the no-CM group (24.3% vs. 15.6%, *p* < 0.001), as was the use of snus (17.6% vs. 13.3%, *p* < 0.001). These findings were also evident when considering frequency. Daily or near-daily smoking was reported by 14.9% of participants in the CM group vs. 8.1% in the no-CM group, and daily snus use was also higher in the CM group (14.7% vs. 10.7%). The group differences remained significant after adjusting for sociodemographic variables. In adjusted models, CM was associated with significantly higher odds of both smoking [odds ratio (OR) 1.65; 95% CI 1.47, 1.86; *p* < 0.001] and snus use (OR 1.36; 95% CI 1.19, 1.56; *p* < 0.05, Table 3).

**Table 2:** Comparison of alcohol and nicotine use by childhood maltreatment (CM) status.

Variable	No CM (*N* = 16 492)	CM (*N* = 1,842)	*p*-value
Alcohol use (*N* = 18 216)			
Alcohol use last 12 months	13 716 (83.7)	1,503 (82.2)	0.095
Frequency of alcohol use last 12 months			
- Never	2,671 (16.3)	326 (17.8)	
- Occasionally	13 122 (80.1)	1,439 (78.7)	0.247
- Daily or near daily	594 (3.6)	64 (3.5)	
AUDIT-C gender-specific thresholds^a^	5,193 (31.7)	610 (33.4)	0.148
AUDIT-C ≥8	548 (3.3)	68 (3.7)	0.402
Alcohol binge weekly or more (*N* = 18 106)	560 (3.4)	71 (3.9)	0.301
Nicotine use			
Nicotine use last 12 months (*N* = 18 214)	4,189 (25.6)	656 (35.8)	< 0.001
Smoking last 12 months (*N* = 18 276)	2,566 (15.6)	446 (24.3)	< 0.001
- Never	13 876 (84.4)	1,388 (75.7)	
- Occasionally	1,230 (7.5)	173 (9.4)	< 0.001
- Daily or near daily	1,336 (8.1)	273 (14.9)	
Snus use last 12 months (*N* = 18 245)	2,180 (13.3)	323 (17.6)	< 0.001
- Never	14 233 (86.8)	1,509 (82.4)	
- Occasionally	429 (2.6)	53 (2.9)	< 0.001
- Daily or near daily	1,751 (10.7)	270 (14.7)	

Values are given as *n* (%). ^a^Gender-specific cut-offs for the AUDIT-C sum score were five for men and four for women.

**Table 3:** Adjusted odds ratios (ORs) from logistic regression analyses predicting alcohol consumption, smoking, and snus use.

Variable	Alcohol use, gender-specific AUDIT-C^a^	Alcohol use, AUDIT-C ≥8	Binge drinking weekly or more	Smoking	Snus use
Child maltreatment	0.99 (0.89, 1.10)	1.14 (0.88, 1.48)	1.21 (0.94, 1.56)	1.65 (1.47, 1.86)^**^	1.36 (1.19, 1.56)^**^
Age	0.98 (0.97, 0.98)^**^	0.97 (0.96, 0.97)^**^	0.99 (0.98, 0.99)^**^	0.99 (0.99, 0.99)^**^	0.95 (0.95, 0.95)^**^
Female sex	0.95 (0.89, 1.02)	0.25 (0.21, 0.30)^**^	0.29 (0.24, 0.34)^**^	0.92 (0.85, 1.00)	0.43 (0.39, 0.47)^**^
Education level^b^	0.82 (0.77, 0.87)^**^	0.63 (0.53, 0.75)^**^	0.80 (0.67, 0.94)^*^	0.48 (0.44, 0.52)^**^	0.87 (0.80, 0.96)^*^

Values are given as ORs and 95% confidence intervals. Child maltreatment was adjusted for age, sex, and education.

^a^Gender-specific cut-offs for the AUDIT-C sum score were 5 for men and 4 for women.

^b^OR for education refers to bachelor level or higher.

^*^p < 0.01,

^**^p < 0.001.

As shown in Table 2, there were no significant group differences in alcohol use between the CM and no-CM groups in the past 12 months. This finding held true across various categorizations of alcohol use, including any use, frequency of use, AUDIT-C cut-off score (traditional gender-specific cut-off or stricter cut-off of 8), and binge drinking. Multivariable logistic regression analyses adjusted for age, sex, and educational status confirmed the absence of significant group differences in the likelihood of exceeding the AUDIT-C cut-offs or reporting weekly binge drinking (Table 3).

**Figure 1:**
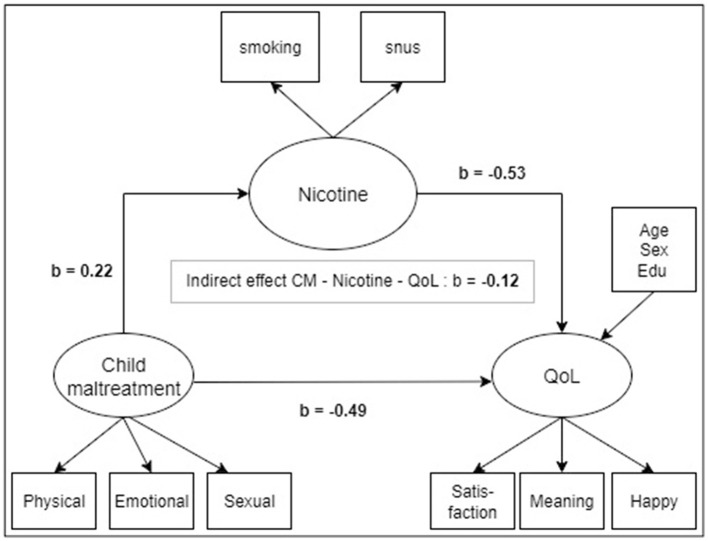
Latent regression model examining the associations between childhood maltreatment (CM), nicotine use, and quality of life (QoL).The model shows the unstandardized beta coefficients between the structural latent constructs in the model. Sociodemographic variables were incorporated as covariates on QoL.

As no significant associations were found between CM and alcohol consumption, alcohol use was excluded from subsequent analyses. The latent regression analysis (Figure 1) confirmed the findings from the separate logistic regression models for smoking and snus, showing that CM was a significant predictor of nicotine use (β = 0.22; 95% CI 0.16, 0.28; *p* < 0.001). Nicotine use, in turn, was negatively associated with QoL (β = −0.53). The total association between CM and QoL was −0.61 (95% CI –0.71,−0.51; *p* < 0.001), including an indirect association involving nicotine use (β = −0.12; 95% CI −0.16,−0.07; *p* < 0.001). The model fit was acceptable (RMSEA = 0.07; CFI = 0.92). Parameter estimates for the full measurement model are shown in the Supplementary File.

## 4 Discussion

CM was reported by 1 in 10 individuals in the sample. Nicotine use was significantly more prevalent among individuals with a history of CM, with differences remaining significant after adjustment. No significant differences were found in alcohol use when comparing individuals with and without a history of CM, including the frequency of use, AUDIT-C scores, and binge drinking. Latent regression analysis showed that CM had a negative association with QoL, including a mediated association involving nicotine use.

The prevalence of the specific categories of CM used in this study was on the lower end or even below the lowest international estimates, which are 6.7% for physical abuse, 6.2% for emotional abuse, and 2.6% for sexual abuse (2, 3). The numbers are also lower than what was found in a recent Norwegian study, which reported rates of 19% for physical abuse, 18% for emotional abuse, and 6% for sexual abuse (3). However, the latter study included negative experiences with “someone at home” when assessing physical and emotional abuse and adults in general when investigating sexual abuse, whereas the present study focused on primary caregivers. Though caution is warranted when interpreting prevalence studies due to methodological differences, such as variations in definitions and question-wording across studies, the present findings suggest that this region in Norway may have a comparatively lower prevalence of CM (2, 41, 42). Nonetheless, even the lowest prevalence, 1.6% for sexual abuse, cannot be regarded as a minor issue. Given the sensitive nature of CM, prevalence estimates are likely underestimated. Even at this level, such a prevalence represents a substantial number of individuals, likely with long-lasting consequences for health and well-being (1, 31, 43).

Our results support previous reports of an association between ACEs and both tobacco smoking and use of smokeless tobacco (9, 44). Notably, the association between ACEs and smoking was found in the present population even with tobacco consumption habits having changed in the past few decades, with a prevalence of daily smoking declining from rates exceeding 40% to less than 10% today (23). Our study further suggests that CM is also a relevant risk factor for snus use, paralleling its association with smoking (45, 46). Why ACEs are associated with use of nicotine was not investigated in the present study. However, nicotine has documented psychoactive benefits in affect regulation, and it has been suggested that people who have experienced ACEs may find it beneficial to use nicotine for mood regulation (47). The stress-sensitization model suggests that individuals with a history of ACEs have greater stress-reactivity and, therefore, are more sensitive to stress later in life, which, again, may lead some to seek relief through substance use (48).

Contrary to our expectations, the study did not reveal a significant association between ACEs and problematic alcohol use. Although previous research has generally found a strong link between ACEs and problematic alcohol consumption (9), the present results are more in line with recent studies reporting no association between ACEs and alcohol consumption (12, 13). The lack of association may be due to alcohol use being normative and common within the studied populations (13). The Norwegian drinking context is characterized by strong regulatory frameworks, relatively low average consumption (in a European context), and episodic high-intensity (binge) weekend drinking in parts of the population (49, 50). Drinking patterns in Norway have increasingly shifted toward more “continental” patterns (50). Nonetheless, we cannot rule out that cultural characteristics may have contributed to weak associations between CM and AUDIT-C scores. To address this possibility, we applied a stricter definition of problematic alcohol use (AUDIT-C cut-off and binge drinking), but this also failed to reveal a significant association.

As anticipated, we found a significant negative association between childhood physical, emotional, or sexual abuse and QoL. These findings are consistent with prior research linking CM to reduced QoL (7). Previous studies have also independently associated smoking, snus use, and alcohol consumption with lower QoL (7, 51–53). Findings from our statistical mediation analysis suggest that nicotine use may partially account for the association between CM and reduced QoL. Low QoL and depression have been associated with a greater likelihood of initiating smoking and a lower likelihood of quitting successfully (51). At the same time, diseases known to be attributable to smoking are independently linked to reduced QoL (16, 54–57). Thus, the association between tobacco product use and QoL is complex and likely bi-directional, with uncertain temporal dynamics that warrant further investigation. Snus has been promoted as a harm-reduction strategy in tobacco control (58), but growing evidence points to negative effects of snus as well, with studies linking it to chronic diseases that are associated with reduced QoL (20, 59). Given increasing consumption and the potential harmful effects of snus use, it is important to examine its link with ACEs (19, 20). It is essential to recognize the evolving patterns of tobacco product use and to ensure that emerging products of growing concern are incorporated into relevant guidelines and recommendations. Although descriptive analyses showed higher snus use among individuals with CM, snus use was not modeled separately in relation to QoL, as described in the Statistical Analysis section. The results are therefore framed as highlighting the complexity of nicotine use patterns in this population. In relation to the ongoing debate on snus as a potential harm-reduction strategy, the present study does not provide empirical data to evaluate or support this hypothesis (58).

With respect to implications, particularly for tobacco-control strategies, we do not suggest targeted interventions directed specifically at individuals with CM histories, as such approaches could risk unintended stigmatization. Rather, our findings highlight the importance of interventions aimed at improving QoL among individuals with a history of CM, including attention to nicotine use within broader health and wellbeing frameworks. Accordingly, we focus on implications at the level of practitioner and public-health awareness, emphasizing the relevance of trauma-informed approaches when addressing nicotine use, without singling out individuals with CM as a distinct subgroup.

### 4.1 Methodological considerations

The relatively low response rate (32%) in this study may have contributed to an underestimation of prevalence. This applies both to the predictor of this study (CM) and the intermediate outcome (substance use), as individuals with more problematic lives are less likely to participate in surveys (60–62). Using retrospective self-reports to assess sensitive experiences, such as CM, carries a risk of false negatives, as individuals may choose not to disclose their experiences or may have forgotten them (63). In contrast, false-positive reports are considered rare (64). Therefore, this approach may miss individuals who feel the topic is too sensitive to report or who have repressed or lost memories of their CM experiences, leading to potential underreporting and misclassification, particularly with respect to prevalence estimates. Thus, such estimates should be interpreted with caution due to potential nonresponse bias. The methodological decision to exclude respondents who selected only “other CM or prefer not to specify” introduced a small amount of additional missingness.

In the present study, alcohol use was assessed using the original version of the AUDIT-C, and binge drinking was operationalized using the third AUDIT-C item, which assesses the frequency of consuming six or more standard alcohol units on one occasion, without a specified timeframe. We note this as a limitation, as binge drinking is commonly defined as a pattern of alcohol consumption that rapidly raises blood alcohol concentration, typically over a short period (e.g., within 2 h). Related to the assessment of negative childhood exposures, a more detailed measure, including additional subtypes or expanded items, could potentially have provided broader coverage. We note that the three CM subtypes included in the latent construct correspond to the core maltreatment dimensions used in the original ACE framework and represent direct (“active”) forms of maltreatment, which were the focus of the present study. We therefore did not include other ACEs from the original ACE questionnaire that reflect more indirect indicators, such as various forms of family dysfunction (e.g., parental separation/divorce or substance use among caregivers). While such factors may create contexts in which maltreatment occurs, they do not constitute direct maltreatment *per se*.

Parental smoking or use of snus is associated with adolescent smoking and use of snus, and there is an association between ACEs and childhood exposure to tobacco use by adults (65, 66). Our study did not take into account whether participants had been exposed to tobacco use by adults during childhood. We note that although the study is cross-sectional, CM was assessed retrospectively, meaning that the exposure precedes the outcomes conceptually. However, this temporal ordering is not sufficient to support causal conclusions. While prevalence estimates may be subject to uncertainty due to potential issues with sample selection and survey methodology, findings from analyses of associations within the dataset are generally considered less sensitive to selection bias than absolute prevalence estimates (67). Although the study included descriptive estimates of CM prevalence, the primary analytical focus was on associations between CM and adult outcomes, motivating a parsimonious analytical approach. Within this framework, nonresponse weighting was not applied in the present analyses. Consistent with this approach, alcohol was excluded from the latent regression analysis, as it did not show meaningful associations in the descriptive analyses and its inclusion would not have contributed substantive information or altered the overall interpretation. The large sample size drawn from a general population remains a strength of the study.

## 5 Conclusion

The findings underscore the association between CM and increased nicotine use, including both smoking and snus, whereas alcohol use patterns appear unaffected. The observed indirect association between CM and reduced QoL involving nicotine use suggests that tobacco-related behaviors may partially account for this association. These findings highlight the need for targeted interventions to improve QoL among individuals with a history of CM, including interventions addressing nicotine use.

## Data Availability

The original contributions presented in the study are included in the article/Supplementary material, further inquiries can be directed to the corresponding author.
