# Supplementary material for: Child maltreatment and substance use: associations with alcohol use, nicotine use, and quality of life in a Norwegian population sample
Source: Front Public Health. 2026 May 15;14:1821179. doi: 10.3389/fpubh.2026.1821179 (PMC13219326; doi:10.3389/fpubh.2026.1821179)
Supplement: Supplementary file 1 [file Supplementary_file_1.docx]

**Supplementary file**

Parameter estimates for the full measurement model are shown in Figure 2 (unstandardized coefficients) and Figure 3 (standardized coefficients).


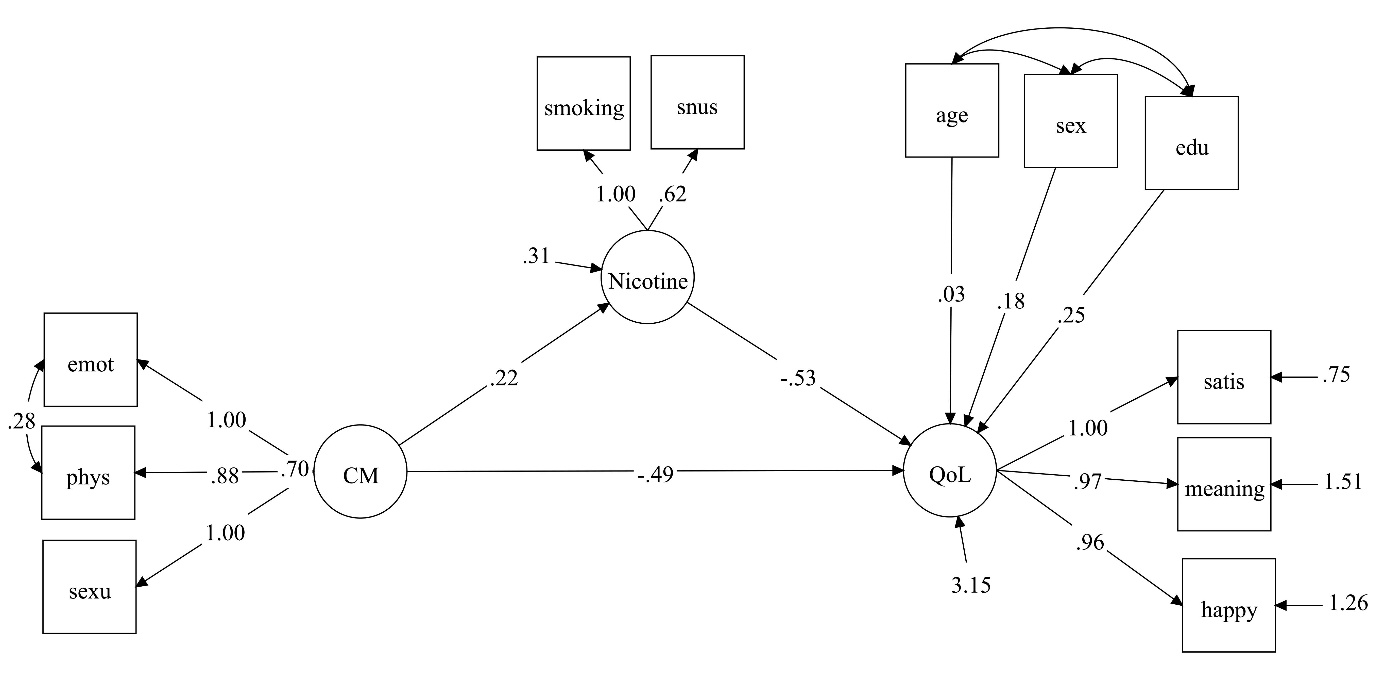


**Figure 2.** Latent regression model with *unstandardized beta coefficients* examining associations between childhood maltreatment (CM), nicotine use, and quality of life (QoL). Abbreviations: Emot = Emotional abuse, phys = physical abuse/violence, sexu = sexual abuse, edu = educational attainment, satis = satisfaction, meaning = meaningfulness, happy = happiness.

**
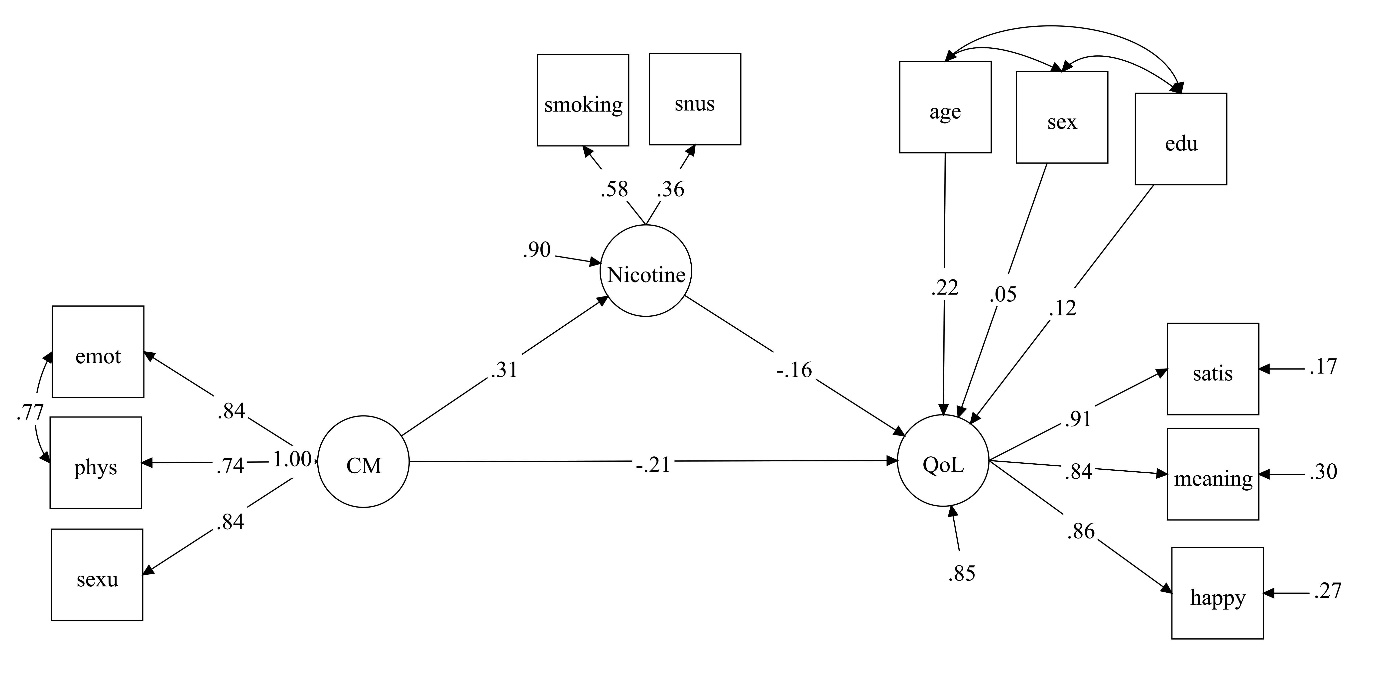
**

**Figure 3.** Latent regression model with *standardized beta coefficients* examining associations between childhood maltreatment (CM), nicotine use, and quality of life (QoL). Abbreviations: Emot = Emotional abuse, phys = physical abuse/violence, sexu = sexual abuse, edu = educational attainment, satis = satisfaction, meaning = meaningfulness, happy = happiness.
